# High-Performance Multi-Level Inverter with Symmetry and Simplification

**DOI:** 10.3390/mi15060766

**Published:** 2024-06-07

**Authors:** Jenn-Jong Shieh, Kuo-Ing Hwu, Sheng-Ju Chen

**Affiliations:** 1 Department of Electrical Engineering, Feng Chia University, No. 100, Wenhwa Road, Seatwen, Taichung 40724, Taiwan; jjshieh@fcu.edu.tw; 2 Department of Electrical Engineering, National Taipei University of Technology, No. 1, Sec. No. 3, Zhongxiao E. Rd., Taipei 10608, Taiwan; unlimitedrulebook666@gmail.com

**Keywords:** double voltage clamp, inverter, seven levels, single phase, symmetric

## Abstract

This paper presents a high-performance, multilevel inverter with symmetry and simplification. This inverter is a single-phase, seven-level symmetric switched-capacitor inverter based on the concept of the double voltage clamping circuit connected to the half-bridge circuit. Above all, only a single DC power supply is used to achieve a single-phase inverter with seven levels and a voltage gain of three. In addition to analyzing the operating principle of the proposed switched-capacitor multilevel inverter in detail, the stability analysis and controller design are carried out by the state-space averaging method. The feasibility and effectiveness of the proposed structure are validated by some simulated results based on the PSIM simulation tool and by some experiments using FPGA as a control kernel, respectively. However, in this study, the switches were implemented by MOSFETs to meet a high switching frequency. These MOSFETs can be replaced by IGBTs in the motor drive applications so that the used switching frequency can be reduced.

## 1. Introduction

Due to the limitation of fossil fuels and their non-renewable nature after combustion, sustainable energy has become a major topic of discussion in today’s society. Sustainable oil and coal are decreasing year by year, while the amount of renewable energy is increasing year by year, with hydroelectricity being the most important type of renewable energy. Meanwhile, solar power and biomass power generation are also increasing year by year [1].

Renewable energy can help promote the decentralization of energy and electricity and enhance the security and reliability of energy supply. However, the development of renewable energy still faces a number of challenges, such as energy storage and transmission technologies, energy policies, and market uncertainties. Therefore, in the process of promoting the development of renewable energy, it is necessary to strengthen technological research and development and market development and actively formulate and implement policies and regulations favorable to the development of renewable energy. The American Institute of Electrical and Electronics Engineers (Institute of Electrical and Electronics Engineers, IEEE) developed the IEEE 1547 standard [2] is an interconnection standard for distributed energy resources, including solar photovoltaic systems, wind turbines, fuel cells, etc. The standard includes many requirements, such as AC power control, frequency and voltage control, protection and safety, fault and failure mode management, etc. The IEEE 519 standard [3] proposes to protect the equipment in the power system from the adverse effects of harmonics and to protect the harmonic content in the system from exceeding the safe and acceptable limits. In addition, recommendations are provided on the limitation and control of harmonic voltages and currents in power systems. In Europe, the International Electrotechnical Commission (IEC) has issued the IEC 60038 standard [4], which defines the range of voltages and frequencies to be supplied to power systems used at fixed frequencies to ensure compatibility between different types of electrical equipment and stability and reliability under different environmental conditions. At the same time, a series of IEC 61000 standards [5] was promulgated under the title electromagnetic compatibility (EMC). Among these standards, the IEC 61000-3-X standard covers several aspects, including output and input noise limitations for electrical and electronic equipment in power systems, limitations on harmonics and abnormal voltages, etc. Among these, IEC 61000-3-2 [5] aims to limit the effect of electrical equipment (e.g., household appliances, industrial equipment, etc.) on harmonic currents in the public low-voltage power supply network, and IEC 61000-3-3 [6] aims to determine the limits of the input noise of electronic equipment in low-voltage power systems in order to ensure the compatibility of the various types of electronic equipment and to minimize the disturbances and damage in the power grid. In addition, the IEC 62109-1 standard [7] has been developed for the specification of safety requirements for inverters in solar power systems. 

Currently, the electricity provided by renewable energy can be categorized as an alternating current (AC) through rotary motors for hydropower and wind power or a direct current (DC) through semiconductor physical and chemical energy conversion for solar power and fuel cells. In order to cope with the AC system of utility, the AC/DC converter combined with the DC/AC converter or DC/AC converter can be used to convert the green energy to AC power. Therefore, the DC/AC converter is one of the most important technologies used for power transmission.

Nowadays, the development of DC–AC converters tends to comprise high efficiency and low harmonic components; therefore, the purpose of the development trend of a multilevel inverter (MLI) is to achieve a large number of levels with a small number of power components to minimize the component loss. The principle of the multilevel inverter is to utilize several capacitors and power switches to change the conduction path to clamp and divide the voltage of several levels so as to make the output voltage present at several levels. The more levels there are, the more the output voltage can reduce the multiple harmonic components of the output waveform without the need for external inductors or filters, and the output voltage can be close to the sinusoidal waveform. In addition, the voltage on part of the switches can be clamped by the level voltage to reduce the voltage stress on them and improve the overall output efficiency. The literature [8] discusses the operating principles of classical multilevel inverter circuits and how to extend the inverter topology to increase the number of levels, including a neutral point clamped inverter (NPC), flying capacitor inverter (FC), and cascaded H-bridge converter (CBC), cascaded H-Bridge converter (CHB), and so on.

**Figure 1:**
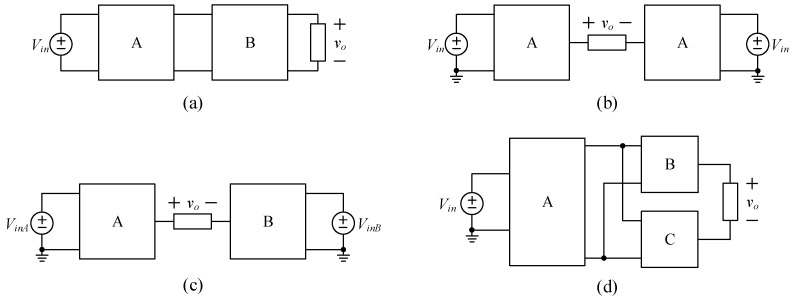
Four types of increasing numbers of voltage levels: (**a**) cascaded; (**b**) symmetrical; (**c**) asymmetrical; and (**d**) hybrid.

As shown in Figure 1, considering the development cost and component selection of multilevel inverters, the output voltage quality can be improved through several modular circuits. This figure discusses four ways to increase the number of voltage levels of multilevel inverters. Circuits A, B, and C can be multilevel inverters, half-bridge or full-bridge circuits composed of switches or switched-capacitor circuits composed of capacitors and switches.

In Figure 1a, the cascaded type is the output of circuit A connected with the output of circuit B, and the system output comes from circuit B. In [9], a module consisting of two independent voltage sources and two power switches is used as the input voltage source of a conventional full-bridge, where the input voltage of the full-bridge is varied by switching the power switches to achieve a five-level voltage output. Study [10] continues the concept of study [9] by combining a boost converter with a diode voltage doubling circuit to boost a single voltage source into a DC voltage with several voltage levels, which can be supplied to the conventional full-bridge for the multilevel AC voltage output. The literature [11] consists of two sets of three-level diode neutral point clamped (DNPC) circuits, where the output of the first set is connected to the neutral point of the second set to achieve a seven-level voltage output and to minimize the impact of the load on the neutral point of voltage. The literature [12] proposes a seventeen-level voltage output, which is characterized by replacing part of the half-bridge circuit in the active neutral point clamped (ANPC) circuit with a high-order flying capacitor (FC) circuit to reduce the majority of the voltage stresses on the power switches to 1/16 of the input voltage. The circuit structures shown in [9,10,11,12] can construct the output capability of circuit B based on the output characteristics of circuit A. However, as the number of levels increases, the number of components increases, leading to an increase in the conduction losses in the circuit.

In Figure 1b, the symmetric type is two identical circuits connected together, and the system output comes from their individual terminals. In [13,14], two sets of three-level DNPC circuits were connected in a pair to achieve a five-level voltage output. In addition, the neutral points of the two identical circuits are connected to the neutral points of the two capacitors, and the current flow to the neutral point of the two circuits was canceled by the unipolar pulse width modulation (PWM) method to solve the neutral point voltage offset problem of the triple-neutral point clamped circuit with the space vector control used to minimize the neutral-point voltage offset. The literature [15] considers the problem of the conduction loss caused by the forward bias of the diode when the DNPC circuit outputs a high current, while a three-level T-type neutral point clamped circuit can be used to improve the conduction loss in the DNPC circuit in [13], instead of this, the voltage stresses on some power switches are increased. Study [16] is based on study [15] using two independent power sources with two switches cross-connected to the two half-bridges to achieve seventeen levels of voltage output by switching the cross-connected switches. In [17], a three-phase F-type neutral point clamping circuit is proposed to improve the total standing voltage (TSV) without increasing the number of components in the T-type circuit, thereby taking into consideration the component selection and cost, and a three-phase system is constructed using three sets of the same circuits, which achieves a five-level line-to-line voltage output. By analyzing the DNPC and T-type circuits above, it can be seen that all the power switches of the former are clamped by the neutral voltage, thereby resulting in the voltage stress being half of the input voltage, which has better-switching characteristics and its output specification tends to be high-voltage with a low-current output, while the latter has a smaller number of components, thereby resulting in a reduction in the total voltage drop at turn-on, which has better turn-on characteristics and its output specification tends to be low-voltage with a high-current output. As for the F-type, its output voltage and current specifications are in between the above two circuits, so it is easier to achieve maximum efficiency when operating at a medium load. In [13,14,15,16], two sets of the same circuit topology are independently controlled so that the output voltage levels are the sum of the levels of the two circuits, and the control complexity can be reduced by the same control strategy. However, two independently controlled circuits mean that more than two power switches are turned on and turned off at the same time, thereby leading to an increase in switching loss.

In Figure 1c, the asymmetric type is two different circuits connected together, and the system output comes from their individual terminals. Study [18] discusses how to reduce the number of components to minimize the total harmonic distortion (THD) by constructing the multilevel inverter asymmetrically. Study [19] presents a classic totem pole structure with power factor correction (PFC), where a power switch is used instead of a diode to realize the bidirectional operation of the converter. In particular, the control strategy of the circuit adopts a mix of high and low speeds to drive two switching circuits: one is a low-speed arm half-bridge, which uses power switches with low on-resistance to switch between positive and negative half-cycles of the sinusoidal waveform, and the other is a high-speed arm, which uses a multilevel inverter circuit equipped with a low-voltage stress feature to switch between high-frequency switches, so as to reduce the THD. Therefore, the switching characteristics of the two sets of switching circuits can be distinguished, and accordingly, by differentiating the switching characteristics of the two switching circuits, the power switches can be suitably selected to achieve high efficiency. The references [20,21,22] present DNPC, T-type, and F-Type circuits, respectively. For these circuits, adding a half-bridge circuit for low-speed control and a PWM control for mixing high and low speeds to a three-level neutral point clamping circuit achieves a five-level voltage output. Compared with the symmetrical structure proposed in studies [13,15,17], the totem pole structure can achieve the same voltage output with fewer components and power loss; however, this structure loses the neutral current cancellation feature and increases the neutral voltage offset. Study [23] uses a T-type circuit with low on-state resistance for c and a DNPC circuit with low voltage stress for high-speed control to achieve a five-level voltage output and reduce the neutral voltage offset by sharing the neutral point. In study [24], a three-level flying capacitor circuit is added to a low-frequency switching half-bridge circuit to construct a totem pole structure to achieve five-level voltage output, in which the flying capacitor is self-balanced by high-frequency charging and discharging based on the output current so that the problem of the neutral voltage can be completely solved. Flying capacitor circuits require additional pre-charging or auxiliary circuits to ensure that the voltages across the power switches can be reduced in advance before the circuit operates. The circuit structures shown in [19,20,21,22,23,24] are based on the difference in the characteristics of circuits A and B to create an additional voltage hierarchy. However, the different characteristics of circuits A and B increase the complexity of the control strategy, and the voltage gains of circuits A and B need to be considered to match each other.

In Figure 1d, the hybrid type is three circuits connected together. Circuit B is paralleled with circuit C at their input sides. After this, the output of circuit A is connected with these input sides. Finally, the system output comes from the individual terminals of the two circuits, B and C. Studies [25,26] use an asymmetric five-level circuit in series with a classical H-bridge circuit to enhance the number of levels and the voltage gain. The five-level circuit mentioned in [25] adopts the asymmetric T-type circuit in [23], while the five-level circuit mentioned in [26] adopts the asymmetric five-level flying capacitor circuit in [24], which is connected to a classical H-bridge circuit to realize the nine-level voltage output. Study [27] follows [23] by using the T-type circuit as the module of the series-connected structure. At the same time, the transformer is used to connect the series-connected module to increase the voltage gain and the number of levels. However, the transformer generates a magnetizing current, leading to an increase in losses, and the neutral point voltage of the T-type circuit varies depending on the load. Study [28] realizes the control of real and virtual power injected into the grid. In addition, the circuit structure must incorporate an independent DC power supply of 0.4 times the input voltage to achieve eleven levels of voltage output from two asymmetric voltage sources. The circuit structures shown in [25,26,27,28] can achieve high levels of the voltage output in more than two ways, as mentioned in Figure 1. Among them, the circuit structure presented in [25,26,28] requires an additional independent power supply to increase the voltage gain, thereby making this circuit structure not applicable to single-input DC voltage source systems.

In this paper, a single-phase seven-level symmetric switched-capacitor inverter is based on the concept of the double voltage clamping circuit connected in series with the half-bridge circuit. Above all, only a single DC power supply is used to achieve a single-phase inverter converter with seven levels and a voltage gain of three. The remainder of this paper is organized as follows: Section 2 introduces the proposed circuit structure of the proposed inverter. Section 3 investigates the basic operating principle of this inverter. Section 4 presents the steady-state and small-single analyses. Section 5 presents the design considerations. Section 6 validates the feasibility and effectiveness of such an inverter by simulated and experimental results, respectively. Finally, Section 7 draws a conclusion.

## 2. Introduction to the Proposed Circuit Structure

**Figure 2:**
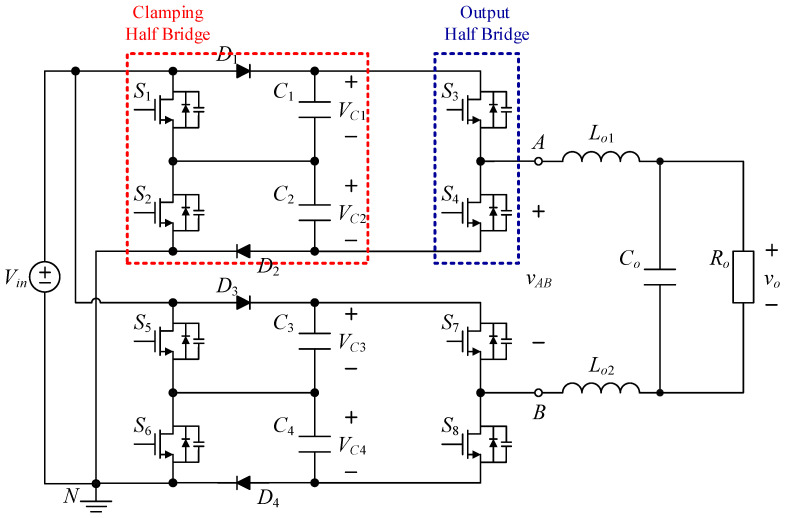
Circuit structure of the proposed DC–AC converter.

The proposed inverter is shown in Figure 2, where switched-capacitor circuits are composed of capacitors and power switches and are often used to maintain the voltages across the capacitors at several times the input DC voltage. The output voltage of the circuit is controlled by switching the active components to charge and discharge the capacitor to achieve a higher output voltage level.

A symmetrical structure multilevel inverter is composed of two sets of identical multilevel inverter circuits in a pairwise manner, and the main difference between the proposed multilevel inverter and the existing multilevel inverter lies in the symmetry of the output structure of the proposed circuit. In general, the existing symmetric multilevel inverter usually has the circuit components with different currents flowing through them between positive and negative half cycles, so different drive control strategies are required, resulting in control difficulty; however, the proposed symmetrical multilevel inverter with currents flowing through the circuit components between positive and negative half-cycles are the same, so only a unipolar drive control strategy is needed to realize the switching control of this inverter, greatly simplifying the system design and control.

The proposed single-phase seven-level symmetric switched-capacitor DC–AC converter is constructed using the concept of two double voltage clamping circuits connected to individual half-bridge circuits. As shown in Figure 2, the output of the half-bridge circuit consisting of two power switches is connected to the load side, which is called the output half bridge; similarly, the double voltage clamping circuit is composed of the half-bridge circuit consisting of two diodes, two capacitors and two power switches, which is called the clamping half bridge.

In addition, due to the symmetry of the output structure of this converter, the currents flowing through the circuit components at positive and negative half-cycles are the same, so only a unipolar drive control strategy is needed, combined with the level shift-pulse width modulation (LS-PWM) to control the whole circuit.

## 3. Operating Principle and Associated Analysis of the Proposed Inverter

The proposed inverter shown in Figure 2 uses eight switches, four diodes, four clamping capacitors, two filter inductors, one output capacitor, and one output resistor. In addition, switches *S*_1_ to *S*_4_ and switches *S*_5_ to *S*_8_ are symmetrical to each other. Therefore, it is only necessary to analyze the operating principle of the positive half-cycle.

### 3.1. Symbol Definitions and Assumptions

Before analyzing the circuit behavior, the used symbol definitions and the required assumptions are as follows:

(1) *V_in_* is the input voltage, *v_o_* is the output voltage, *N* is the reference point of zero potential, and *R_o_* is the output resistor;
(2) *L_o_*_1_ and *L_o_*_2_ are the filter inductors, *C_o_* is the filter capacitor, and *C*_1_ to *C*_4_ are the clamping capacitors;
(3) *i_L_* is the current flowing through the filter inductors *L_o_*_1_ and *L_o_*_2_, *i_Co_* is the current flowing through the filter capacitor *C_o_*, and *i_o_* is the output current;
(4) *S*_1_ to *S*_8_ are the active switches and *D*_1_ to *D*_4_ are the passive switches;
(5) By assuming that all the values of the clamping capacitors are large enough, the voltages across them can be viewed as constant, i.e., *V_C_*_1_ = *V_C_*_2_ = *V_C_*_3_ = *V_C_*_4_ = *V_in_*;
(6) It is assumed that all the components are regarded as ideal.

### 3.2. Operating Principle Analysis

**Figure 3:**
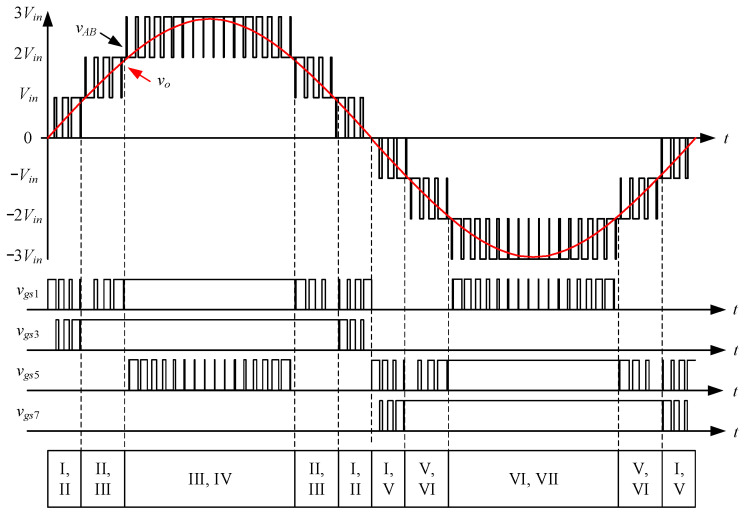
Driving signal waveforms for the switches.

Figure 3 shows the driving signal waveforms for the switches. There are seven states of circuit behavior which correspond to the seven levels of the voltage output. Among them, *v_gs_*_1_ to *v_gs_*_8_ are the driving signals for the switches *S*_1_ to *S*_8_, respectively. As the voltage output operates at a positive half-cycle, the converter operating between states I and II is mainly performed by the switch *S*_3_ to carry out PWM switching to build up the first voltage level, and at this time, the switch *S*_1_ is driven by the complementary signal of *S*_3_, and the switches *S*_5_ and *S*_7_ are kept at normal cutoff; the converter operating between states II and III is mainly performed by the switch *S*_1_ to carry out PWM switching to establish the second voltage level, and at this time, the switch *S*_3_ is kept at normal conduction, while the switches *S*_5_ and *S*_7_ are kept at normal cutoff; the converter operating between states III and IV is mainly performed by the switch *S*_5_, and the same arm switch is driven by inverting the PWM switching of the switch *S*_5_ to establish the third voltage level, and at this time, the switches *S*_1_ and *S*_3_ are kept at normal conduction, while switch *S*_7_ is kept at normal cutoff.

In addition, due to the symmetrical output structure of the converter, the circuit components of the currents flowing through the positive and negative half-cycles of the converter are the same, so only a unipolar drive control strategy is needed, combined with the level shift-pulse width modulation (LS-PWM).

**Figure 4:**
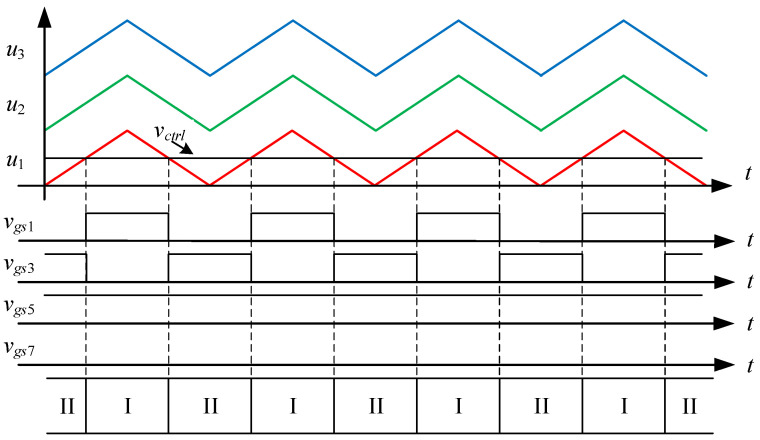
Driving signals for state I.

**Figure 5:**
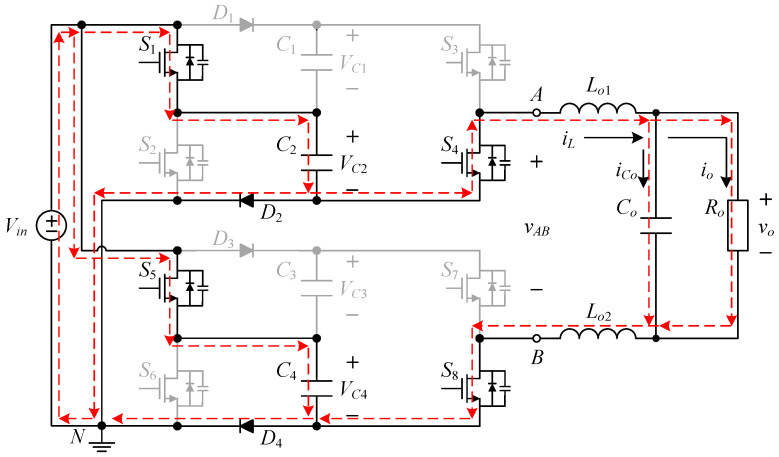
Current flow for state I.

State I. [0 < *v_ctrl_* < *u*_1_]: as shown in Figure 4, the control force *v_ctrl_*, together with triangular waveforms *u*_1_, *u*_2_, and *u*_3_ performs LS-PWM. When the control force *v_ctrl_* is located between 0 and the triangular waveform *u*_1_, the converter enters into state I, as shown in Figure 5. Since the switches *S*_1_, *S*_4_, *S*_5_, and *S*_8_ are on and the switches *S*_2_, *S*_3_, *S*_6_, and *S*_7_ are off, the current *i_L_* flowing through inductors *L_o_*_1_, *L_o_*_2_ forms a loop from the input voltage *V_in_* through the switches *S*_1_, *S*_4_, *S*_5_, *S*_8_, the capacitor *C*_2_, and the diode *D*_4_. Therefore, the voltage between points *A* and *B* is zero, i.e., *v_AB_* = 0.

At state I, according to Kirchhoff’s voltage law (KVL), the following equation can be obtained:

(1) $$v_{L}(t)=L\cdot \frac{di_{L}(t)}{dt}=-3R_{on}\cdot i_{L}(t)-v_{o}(t)+V_{in}-V_{C}-V_{F}$$

At state I, according to Kirchhoff’s current law (KCL), the following equation can be obtained as follows:

(2) $$i_{Co}(t)=C_{o}\cdot \frac{dv_{o}(t)}{dt}=i_{L}(t)-\frac{v_{o}(t)}{R_{o}}$$

where *R_on_* is the conduction resistance of the switches *S*_1_ to *S*_8_, *V_F_* is the forward-biased voltage of the diodes *D*_1_ to *D*_4_, *V_C_* is the voltage of the capacitors *C*_1_ to C_4_, inductance *L* is the sum of inductances *L_o_*_1_ and *L_o_*_2_, and *i_L_*_1_, *i_o_*(*t*) is the current flowing through the load resistor *R_o_*, i.e., $i_{o}(t)=\frac{v_{o}(t)}{R_{o}}$.

After combining Equations (1) and (2), the state equation corresponding to state I can be obtained as follows:

(3) $$\frac{d}{dt}x(t)=A_{1}\cdot x(t)+B_{1}\cdot U$$

where

(4) $$x(t)=\left(\begin{array}{l} i_{L}(t)\\ v_{o}(t) \end{array}\right)$$

(5) $$U=\left(\begin{array}{l} V_{in}\\ V_{F}\\ V_{C} \end{array}\right)$$

(6) $$A_{1}=\left(\begin{array}{cc} \frac{-3R_{on}}{L} & \frac{-1}{L}\\ \frac{1}{C_{o}} & \frac{-1}{R_{o}C_{o}} \end{array}\right)$$

(7) $$B_{1}=\left(\begin{array}{ccc} \frac{1}{L} & \frac{-1}{L} & \frac{-1}{L}\\ 0 & 0 & 0 \end{array}\right)$$

**Figure 6:**
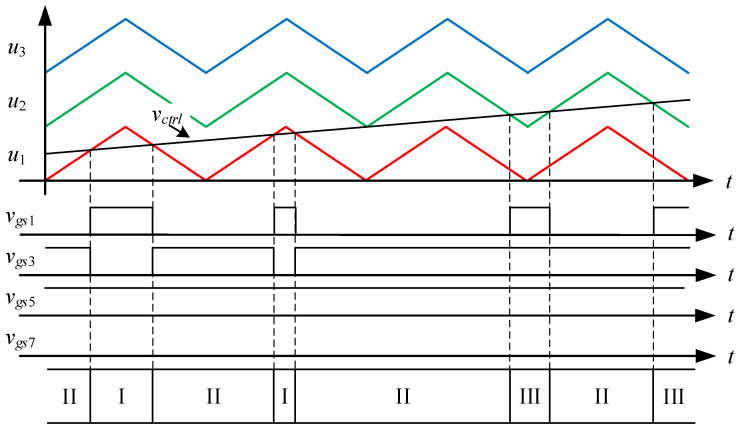
Driving signals for state II.

**Figure 7:**
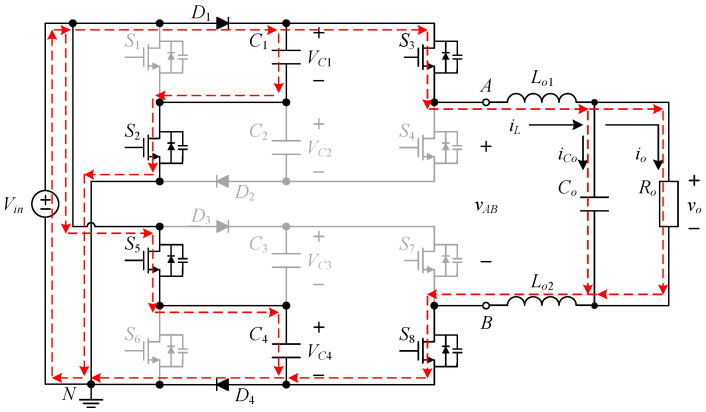
Current flow for state II.

State II. [*u*_1_ < *v_ctrl_* < *u*_2_]: As shown in Figure 6, when the control force *v_ctrl_* is located between the triangular waves *u*_1_ and *u*_2_, the converter enters into state II. As shown in Figure 7, since the switches *S*_2_, *S*_3_, *S*_5_, and *S*_8_ are on and the switches *S*_1_, *S*_4_, *S*_6_, and *S*_7_ are off, the inductor current *i_L_* flows from the input voltage *V_in_* through the switches *S*_3_, *S*_8_, and the diodes *D*_1_, *D*_4_ to form a loop. Therefore, the voltage between points *A* and *B* is the input voltage *V_in_*, i.e., *v_AB_* = *V_in_*.

At state II, according to Kirchhoff’s voltage law (KVL), the following equation can be obtained:

(8) $$v_{L}(t)=L\cdot \frac{di_{L}(t)}{dt}=-2R_{on}\cdot i_{L}(t)-v_{o}(t)+V_{in}-2V_{F}$$

At state II, according to Kirchhoff’s current law (KCL), the following equation can be obtained:

(9) $$i_{Co}(t)=C_{o}\cdot \frac{dv_{o}(t)}{dt}=i_{L}(t)-\frac{v_{o}(t)}{R_{o}}$$

After combining Equations (8) and (9), the state equation corresponding to state II can be obtained as follows:

(10) $$\frac{d}{dt}x(t)=A_{2}\cdot x(t)+B_{2}\cdot U$$

where

(11) $$A_{2}=\left(\begin{array}{cc} \frac{-2R_{on}}{L} & \frac{-1}{L}\\ \frac{1}{C_{o}} & \frac{-1}{R_{o}C_{o}} \end{array}\right)$$

(12) $$B_{2}=\left(\begin{array}{ccc} \frac{1}{L} & \frac{-2}{L} & 0\\ 0 & 0 & 0 \end{array}\right)$$

**Figure 8:**
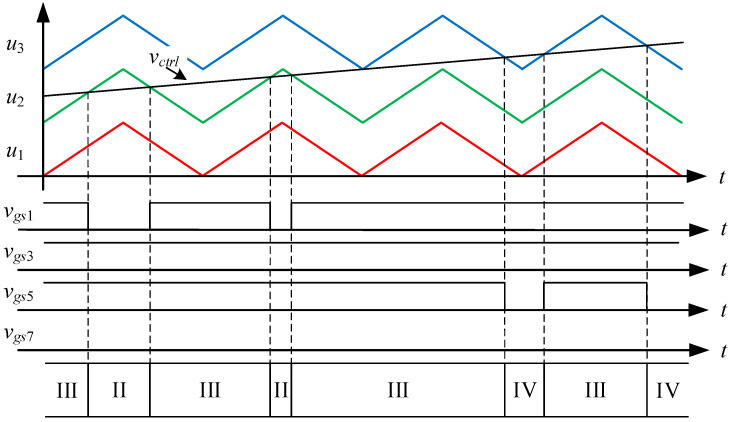
Driving signals for state III.

**Figure 9:**
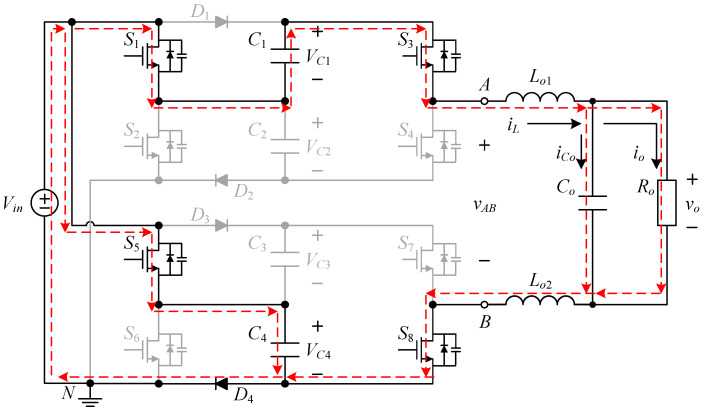
Current flow for state III.

State III. [*u*_2_ < *v_ctrl_* < *u*_3_]: As shown in Figure 8, the converter enters state III when the control force *v_ctrl_* is located between the triangular waveforms *u*_2_ and *u*_3_. As shown in Figure 9, since the switches *S*_1_, *S*_3_, *S*_5_ and *S*_8_ are on and the switches *S*_2_, *S*_4_, *S*_6_ and *S*_7_ are off, the inductor current *i_L_* flows from the input voltage *V_in_* through the switches *S*_1_, *S*_3_, *S*_5_, *S*_8_, the capacitor *C*_1_ and the diode *D*_4_ to form a loop. Therefore, the voltage between points *A* and *B* is double the input voltage *V_in_*, i.e., *v_AB_* = 2 *V_in_*.

At state III, according to Kirchhoff’s voltage law (KVL), the following equation can be obtained:

(13) $$v_{L}(t)=L\cdot \frac{di_{L}(t)}{dt}=-3R_{on}\cdot i_{L}(t)-v_{o}(t)+V_{in}+V_{C}-V_{F}$$

At state III, according to Kirchhoff’s current law (KCL), the following equation can be obtained:

(14) $$i_{Co}(t)=C_{o}\cdot \frac{dv_{o}(t)}{dt}=i_{L}(t)-\frac{v_{o}(t)}{R_{o}}$$

After combining Equations (13) and (14), the state equation corresponding to state III can be obtained:

(15) $$\frac{d}{dt}x(t)=A_{3}\cdot x(t)+B_{3}\cdot U$$

where

(16) $$A_{3}=\left(\begin{array}{cc} \frac{-3R_{on}}{L} & \frac{-1}{L}\\ \frac{1}{C_{o}} & \frac{-1}{R_{o}C_{o}} \end{array}\right)$$

(17) $$B_{3}=\left(\begin{array}{ccc} \frac{1}{L} & \frac{-1}{L} & \frac{1}{L}\\ 0 & 0 & 0 \end{array}\right)$$

**Figure 10:**
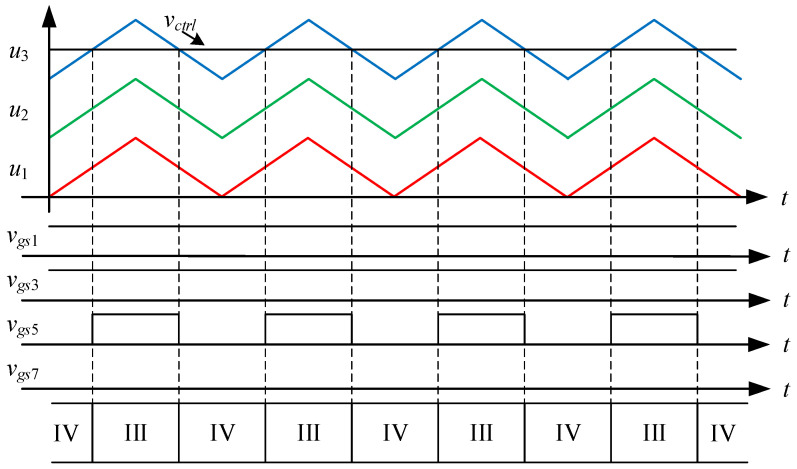
Driving signals for state IV.

**Figure 11:**
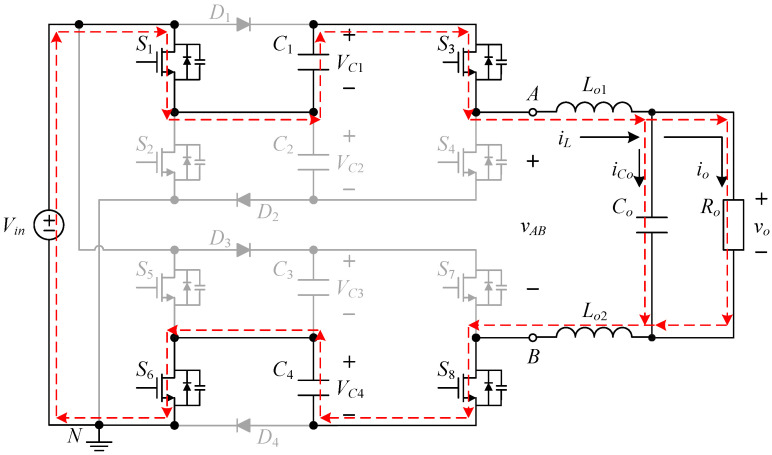
Current flow for state IV.

State IV. [*u*_3_ < *v_ctrl_*]: As shown in Figure 10, when the control force *v_ctrl_* is larger than the triangular wave *u*_3_, the converter enters into state IV. As shown in Figure 11, since the switches *S*_1_, *S*_3_, *S*_6_, and *S*_8_ are on and the switches *S*_2_, *S*_4_, *S*_5_, and *S*_7_ are off, the inductor current *i_L_* forms a loop from the input voltage *V_in_* through the switches *S*_1_, *S*_3_, *S*_6_, *S*_8_, and the capacitors *C*_1_, *C*_4_ form a loop. Therefore, the voltage between points *A* and *B* is triple the input voltage *V_in_*, i.e., *v_AB_* = 3 *V_in_*.

At stage IV, according to Kirchhoff’s voltage law (KVL), the following equation can be obtained:

(18) $$v_{L}(t)=L\cdot \frac{di_{L}(t)}{dt}=-4R_{on}\cdot i_{L}(t)-v_{o}(t)+V_{in}+2V_{C}$$

At state IV, according to Kirchhoff’s current law (KCL), the following equation can be obtained:

(19) $$i_{Co}(t)=C_{o}\cdot \frac{dv_{o}(t)}{dt}=i_{L}(t)-\frac{v_{o}(t)}{R_{o}}$$

After combining Equations (18) and (19), the state equation corresponding to state III can be obtained as follows:

(20) $$\frac{d}{dt}x(t)=A_{4}\cdot x(t)+B_{4}\cdot U$$

where

(21) $$A_{4}=\left(\begin{array}{cc} \frac{-4R_{on}}{L} & \frac{-1}{L}\\ \frac{1}{C_{o}} & \frac{-1}{R_{o}C_{o}} \end{array}\right)$$

(22) $$B_{4}=\left(\begin{array}{ccc} \frac{1}{L} & 0 & \frac{2}{L}\\ 0 & 0 & 0 \end{array}\right)$$

### 3.3. Switch Behavior and Clamping Capacitor Behavior

Table 1 shows the switching behavior and clamping capacitor charging and discharging behavior, in which ‘1’ and ‘0’ represent the switch on and off, respectively, ‘C’ and ‘D’ represent charging and discharging, respectively, and ‘---’ means no action is taken.

## 4. Steady-State Analysis and Small-Signal Analysis

In the following, the quiescent operating point and the small-signal model are discussed.

### 4.1. State-Space Averaging Model

From Figure 4, Figure 6, Figure 8 and Figure 10, it is clear that there are three switching modes when the output voltage operates at a positive half-cycle. The control force *v_ctrl_* is compared with the triangular wave *u*_1_ to make the converter switch between states I and II, and the corresponding duty cycle in state II is *D_a_*; the control force *v_ctrl_* is compared with the triangular wave *u*_2_ to make the converter switch between states II and III, and the corresponding duty cycle in state III is *D_b_*; and the control force *v_ctrl_* is compared with the triangular wave *u*_3_ to make the converter switch between states III and IV, and the corresponding duty cycle in state IV is *D_c_*. Hence, the associated equations for the duty cycles *D_a_*, *D_b_* and *D_c_* are shown below:

(23) $$D_{a}=\frac{v_{ctrl}}{V_{m}},\;0<v_{ctrl}<V_{m}$$

(24) $$D_{b}=\frac{v_{ctrl}}{V_{m}}-1,\;V_{m}<v_{ctrl}<2V_{m}$$

(25) $$D_{c}=\frac{v_{ctrl}}{V_{m}}-2,\;2V_{m}<v_{ctrl}<3V_{m}$$

where *V_m_* is the peak-to-peak value of the triangular waves *u*_1_, *u*_2_, and *u*_3_.

The state-space averaging method [29] is used to obtain the average state matrixes for the three switching modes as shown below:

(26) $$A_{a}=D_{a}\cdot A_{2}+D_{a}^{\prime }\cdot A_{1},\;B_{a}=D_{a}\cdot B_{2}+D_{a}^{\prime }\cdot B_{1}$$

(27) $$A_{b}=D_{b}\cdot A_{3}+D_{b}^{\prime }\cdot A_{2},\;B_{b}=D_{b}\cdot B_{3}+D_{b}^{\prime }\cdot B_{2}$$

(28) $$A_{c}=D_{c}\cdot A_{4}+D_{c}^{\prime }\cdot A_{3},\;B_{c}=D_{c}\cdot B_{4}+D_{c}^{\prime }\cdot B_{3}$$

By rearranging (26) to (28), the corresponding mean state matrices of the three switching modes are shown as follows:

(29) $$A_{a}=\left(\begin{array}{cc} \frac{-(3-D_{a})R_{on}}{L} & \frac{-1}{L}\\ \frac{1}{C_{o}} & \frac{-1}{R_{o}C_{o}} \end{array}\right),\;B_{a}=\left(\begin{array}{ccc} \frac{1}{L} & \frac{-1-D_{a}}{L} & \frac{-1+D_{a}}{L}\\ 0 & 0 & 0 \end{array}\right)$$

(30) $$A_{b}=\left(\begin{array}{cc} \frac{-(2+D_{b})R_{on}}{L} & \frac{-1}{L}\\ \frac{1}{C_{o}} & \frac{-1}{R_{o}C_{o}} \end{array}\right),\;B_{b}=\left(\begin{array}{ccc} \frac{1}{L} & \frac{-2+D_{b}}{L} & \frac{D_{b}}{L}\\ 0 & 0 & 0 \end{array}\right)$$

(31) $$A_{c}=\left(\begin{array}{cc} \frac{-(3+D_{c})R_{on}}{L} & \frac{-1}{L}\\ \frac{1}{C_{o}} & \frac{-1}{R_{o}C_{o}} \end{array}\right),\;B_{c}=\left(\begin{array}{ccc} \frac{1}{L} & \frac{-1+D_{c}}{L} & \frac{1+D_{c}}{L}\\ 0 & 0 & 0 \end{array}\right)$$

### 4.2. Steady-State Analysis

For the steady-state analysis, considering the small ripple approximation method [29], the state vector $x(t)=\left(\begin{array}{l} i_{L}(t)\\ v_{o}(t) \end{array}\right)$ can be rewritten as $X=\left(\begin{array}{l} I_{L}\\ V_{o} \end{array}\right)$, i.e., the differential state vector $\frac{d}{dt}X=0$, and by substituting this result into (32), the relationship between input vector $U=\left(\begin{array}{l} V_{in}\\ V_{F}\\ V_{C} \end{array}\right)$ and state vector $X=\left(\begin{array}{l} I_{L}\\ V_{o} \end{array}\right)$ can be obtained as follows:

(32) $$\frac{d}{dt}X=A\cdot X+B\cdot U=0$$

(33) $$X=-{\left(A\right)}^{-1}\cdot B\cdot U$$

By assuming that the switch is ideal, i.e., *R_on_* = 0, the diode is an ideal diode, i.e., *V_F_* = 0, and the voltages across clamping capacitors are equal to the input voltage *V_in_*. Hence, by substituting (29) to (31) and into (33), the state vectors of the three switching modes, as well as the relationship between the large signals *I_L_* and *V_o_* and the input voltage *V_in_* for the three switching modes can be obtained as follows:

(34) $$I_{L}=\frac{V_{o}}{R_{o}}=D_{a}\cdot \frac{V_{in}}{R_{o}},\;V_{o}=D_{a}\cdot V_{in}$$

(35) $$I_{L}=\frac{V_{o}}{R_{o}}=(1+D_{b})\cdot \frac{V_{in}}{R_{o}},\;V_{o}=(1+D_{b})\cdot V_{in}$$

(36) $$I_{L}=\frac{V_{o}}{R_{o}}=(2+D_{c})\cdot \frac{V_{in}}{R_{o}},\;V_{o}=(2+D_{c})\cdot V_{in}$$

By substituting (23) to (25) into (34) to (36), the relationship between large signals *I_L_* and *V_o_* and the control force *v_ctrl_* is the same for the three switching methods, as shown in (37) and (38):

(37) $$I_{L}=\frac{V_{o}}{R_{o}}=\frac{v_{ctrl}}{V_{m}}\cdot \frac{V_{in}}{R_{o}}$$

(38) $$V_{o}=\frac{v_{ctrl}}{V_{m}}\cdot V_{in}$$

### 4.3. Small-Signal Analysis

Firstly, the disturbance is added to the duty cycle *D_a_* when the output voltage operates at the first voltage level, and the DC and AC components of the state vector and the input vector are defined as follows:

(39) $$\left\{ \begin{array}{l} D(t)=D_{a}+\tilde{d}(t)\\ D^{\prime }(t)=D_{a}^{\prime }-\tilde{d}(t) \end{array}\right.,\;\left\{ \begin{array}{l} x(t)=X+\tilde{x}(t)\\ u(t)=U+\tilde{u}(t) \end{array}\right.$$

By substituting the duty cycle and the disturbance quantity into (26), the average state matrixes *A* and *B* of the small-signal AC model can be obtained as follows:

(40) $$\begin{array}{l} A=D(t)\cdot A_{2}+D^{\prime }(t)\cdot A_{1}=A_{a}+(A_{2}-A_{1})\cdot \tilde{d}(t)\\ B=D(t)\cdot B_{2}+D^{\prime }(t)\cdot B_{1}=B_{a}+(B_{2}-B_{1})\cdot \tilde{d}(t) \end{array}$$

where the matrices *A_a_* and *B_a_* are the DC components of the average state matrices, as shown in (26).

After this, by linearizing (40), the following equation can be obtained:

(41) $$\begin{array}{ll} \frac{d}{dt}(X+\tilde{x}(t))= & \left(A_{a}+(A_{2}-A_{1})\cdot \tilde{d}(t))\cdot (X+\tilde{x}(t)\right)\\ & +(B_{a}+(B_{2}-B_{1})\cdot \tilde{d}(t))\cdot (U+\tilde{u}(t)) \end{array}$$

(42) $$\frac{d}{dt}\tilde{x}(t)=A_{a}\cdot \tilde{x}(t)+((A_{2}-A_{1})\cdot X+(B_{2}-B_{1})\cdot U)\cdot \tilde{d}(t)+B_{a}\cdot \tilde{u}(t)$$

Therefore, in order to obtain the relationship between the disturbance, the input vector change, and the state vectors of the AC components, transforming the state vectors in the time domain to the state vectors in the frequency domain using the Laplace transform of (42) can be achieved.

(43) $$dS\cdot \tilde{x}(s)=A_{a}\cdot \tilde{x}(s)+\left[(A_{2}-A_{1})\cdot X+(B_{2}-B_{1})\cdot U\right]\cdot \tilde{d}(s)+B_{a}\cdot \tilde{u}(s)$$

(44) $$\tilde{x}(s)={\left(dS-A_{a}\right)}^{-1}\cdot \left\{\left[(A_{2}-A_{1})\cdot X+(B_{2}-B_{1})\cdot U\right]\cdot \tilde{d}(s)+B_{a}\cdot \tilde{u}(s)\right\}$$

where

(45) $$ℒ\left\{\frac{d}{dt}\tilde{x}(t)\right\}=dS\cdot \tilde{x}(s),\;dS=\left(\begin{array}{cc} s & 0\\ 0 & s \end{array}\right)$$

Accordingly, the transfer function of the disturbance of the duty cycle to the state vector is shown below:

(46) $$\begin{array}{ll} G_{xd}(s) & ={\frac{\tilde{x}(s)}{\tilde{d}(s)}|}_{\tilde{u}(s)=0}={\left(dS-A_{a}\right)}^{-1}\cdot \left[(A_{2}-A_{1})\cdot X+(B_{2}-B_{1})\cdot U\right]\\ & ={\left(\begin{array}{cc} s+\frac{(3-D_{a})R_{on}}{L} & \frac{-1}{L}\\ \frac{1}{C_{o}} & s+\frac{1}{R_{o}C_{o}} \end{array}\right)}^{-1}\cdot \left(\left(\begin{array}{cc} \frac{-R_{on}}{L} & 0\\ 0 & 0 \end{array}\right)\cdot X+\left(\begin{array}{ccc} 0 & \frac{-1}{L} & \frac{1}{L}\\ 0 & 0 & 0 \end{array}\right)\cdot U\right)\\ & =\left(\begin{array}{l} G_{id}(s)\\ G_{vd}(s) \end{array}\right) \end{array}$$

In (46), since the small-signal equation is the state vector composed of two transfer functions, the transfer function of the duty-cycle disturbance to the output voltage is shown below:

(47) $$G_{vd}(s)=\frac{V_{C}-V_{F}-I_{L}\cdot R_{on}}{s^{2}\cdot L\cdot C_{o}+s\cdot (\frac{L}{R_{o}}+(3-D_{a})R_{on}\cdot C_{o})+(1+\frac{R_{on}}{R_{o}}\cdot L\cdot C_{o})}$$

Similarly, when the output voltage operates at the second and third voltage levels, the individual transfer functions of the duty-cycle disturbance to the output voltage are shown in (49) and (51):

(48) $$\begin{array}{ll} G_{xd}(s) & ={\left(dS-A_{b}\right)}^{-1}\cdot \left[(A_{3}-A_{2})\cdot X+(B_{3}-B_{2})\cdot U\right]\\ & ={\left(\begin{array}{cc} s+\frac{(2+D_{b})R_{on}}{L} & \frac{-1}{L}\\ \frac{1}{C_{o}} & s+\frac{1}{R_{o}C_{o}} \end{array}\right)}^{-1}\cdot \left(\left(\begin{array}{cc} \frac{-R_{on}}{L} & 0\\ 0 & 0 \end{array}\right)\cdot X+\left(\begin{array}{ccc} 0 & \frac{1}{L} & \frac{1}{L}\\ 0 & 0 & 0 \end{array}\right)\cdot U\right) \end{array}$$

(49) $$G_{vd}(s)=\frac{V_{C}+V_{F}-I_{L}\cdot R_{on}}{s^{2}\cdot L\cdot C_{o}+s\cdot (\frac{L}{R_{o}}+(2+D_{b})R_{on}\cdot C_{o})+(1+\frac{R_{on}}{R_{o}}\cdot L\cdot C_{o})}$$

(50) $$\begin{array}{ll} G_{xd}(s) & ={\left(dS-A_{c}\right)}^{-1}\cdot \left[(A_{4}-A_{3})\cdot X+(B_{4}-B_{3})\cdot U\right]\\ & ={\left(\begin{array}{cc} s+\frac{(3+D_{c})R_{on}}{L} & \frac{-1}{L}\\ \frac{1}{C_{o}} & s+\frac{1}{R_{o}C_{o}} \end{array}\right)}^{-1}\cdot \left(\left(\begin{array}{cc} \frac{-R_{on}}{L} & 0\\ 0 & 0 \end{array}\right)\cdot X+\left(\begin{array}{ccc} 0 & \frac{1}{L} & \frac{1}{L}\\ 0 & 0 & 0 \end{array}\right)\cdot U\right) \end{array}$$

(51) $$G_{vd}(s)=\frac{V_{C}+V_{F}-I_{L}\cdot R_{on}}{s^{2}\cdot L\cdot C_{o}+s\cdot (\frac{L}{R_{o}}+(3+D_{c})R_{on}\cdot C_{o})+(1+\frac{R_{on}}{R_{o}}\cdot L\cdot C_{o})}$$

Since the effect of the parameters *R_on_* and *V_F_* can be omitted, (47), (49) and (51) can be unified as follows:

(52) $$G_{vd}(s)=\frac{{\tilde{v}}_{o}(s)}{\tilde{d}(s)}=\frac{V_{in}}{s^{2}\cdot L\cdot C_{o}+s\cdot \frac{L}{R_{o}}+1}$$

**Figure 12:**
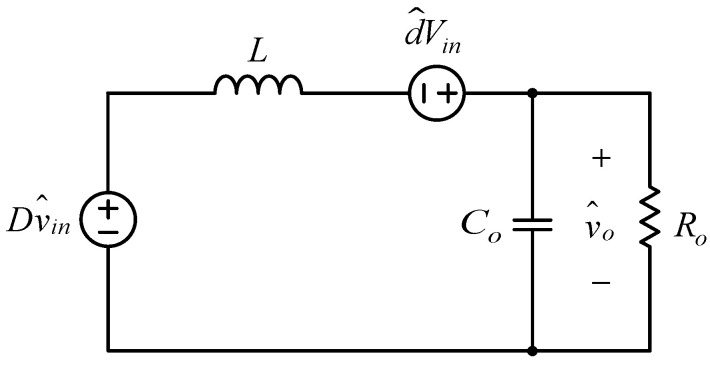
Equivalent small-signal AC model of the proposed inverter.

In other words, from (52), one can see that the equivalent small-signal AC model of the proposed inverter is the same for any state and is shown in Figure 12. From this figure, it can be known that the small-signal AC model of the proposed inverter is the same as that of the conventional DC–DC buck converter. As a result, the control design can be carried out easily and systematically.

## 5. Design Considerations

In the following, the specifications given, the components used, and the controller designed are discussed.

### 5.1. System Configuration

**Figure 13:**
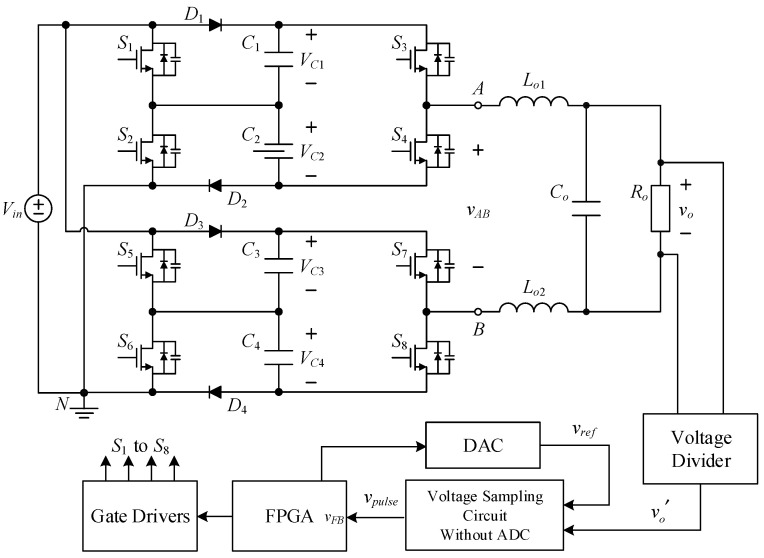
System block diagram of the proposed single-phase switched-capacitor multilevel inverter.

Figure 13 shows the system block diagram of the switching capacitor DC–AC converter proposed in this paper. The circuit system structure consists of the main circuit, the gate drivers, and the output voltage feedback circuit. The operating principle of the main circuit has already been discussed in the previous section, while the gate drivers use UCC21540 isolation drivers. In terms of the voltage feedback circuit, the output voltage *v_o_* is divided into a small voltage signal $v^{\prime }$, the reference voltage *v_ref_* is subtracted from it and modulated into a pulse signal *v_pulse_* using the sampling technique without the analog-to-digital converter (ADC) [30]. Then, the signal *v_pulse_* is sent to the field-programmable gate array (FPGA) controller for conversion into a signal *v_FB_*. After this, the signal *v_FB_* is compensated to obtain gate-driving signals.

**Figure 14:**
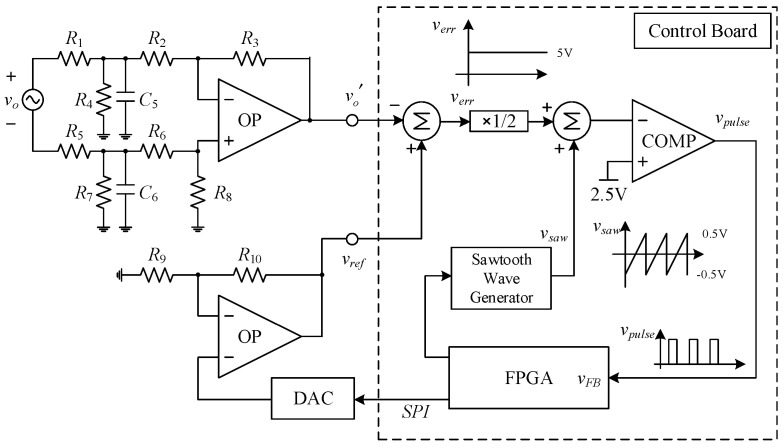
Voltage sampling circuit.

Figure 14 shows how the voltage sampling circuit uses a voltage divider, a differential amplifier, a non-inverting amplifier, and a digital-to-analog converter (DAC). The small voltage signal $v^{\prime }$ is first divided by the voltage divider consisting of resistors *R*_1_, *R*_4_, *R*_5_, and *R*_7_ for the output voltage *v_o_*, and capacitors *C*_5_ and *C*_6_ are added to filter out the high-frequency noise, and then sent to the differential amplifier consisting of resistors *R*_2_, *R*_3_, *R*_6_, and *R*_8_ for the second division. The reference voltage *v_ref_* is amplified by a non-inverting amplifier consisting of resistors *R*_9_ and *R*_10_ to amplify the analog signal from the digital-to-analog converter (DAC). Since the sampling voltage range of the no ADC sampling technique [31] is located between 4 V and 6 V, the reference voltage *v_ref_* is subtracted from the small voltage signal $v_{o}^{\prime }$ to obtain the sampling signal *v_err_*, which is then added with a sawtooth voltage *v_saw_* and a 2.5 V DC voltage, and then passed through a comparator to generate a pulse signal *v_pulse_*. Afterward, this signal is sent to the FPGA controller to be converted into a signal *v_FB_*.

### 5.2. System Specifications

Table 2 shows the specifications of the switching capacitor DC–AC converter proposed in this paper. The converter proposed in this paper adopts the utility voltage provided by the Taiwan Power Company, i.e., the rms output voltage is 110 V, its output frequency is 60 Hz, and its output power is 500 W. The prototype of the converter can be used in the AC power system for mobile lighting equipment and small household appliances. Table 3 shows the components and parameters of the voltage sampling circuit in this paper. 

### 5.3. Controller Design

The control architecture used in this paper is a digital PI controller, where the feedback output voltage is sampled based on no ADC [30], and then the result is fed into the controller to obtain a control force; it is then used by the LS-PWM module to generate the gate drive signals required for power switches.

Firstly, by substituting the relevant values of the components used in the converter in Table 2 into (52), the duty cycle to output voltage transfer function *G_vd_* of the converter can be obtained as follows:

(53) $$G_{vd}(s)=\frac{{\tilde{v}}_{o}(s)}{\tilde{d}(s)}=\frac{58}{s^{2}\cdot 2.794\times 10^{-10}+s\cdot 1.1734\times 10^{-5}+1}$$

Next, the uncompensated loop gain equation is analyzed. Here, *G_vd_* is the transfer function of the analog feedback circuit that converts the output voltage *v_o_* to a small voltage signal $v_{o}^{\prime }$ in the voltage sampling circuit, as shown in Figure 14. Substituting the component values in Table 3 yields the following:

(54) $$\begin{array}{ll} H(s) & =256\times \frac{R_{3}}{R_{2}}\times \frac{{\left({R_{4}}^{-1}+s\cdot C_{5}+{\left(R_{2}+R_{3}\right)}^{-1}\right)}^{-1}}{R_{1}+{\left({R_{4}}^{-1}+s\cdot C_{5}+{\left(R_{2}+R_{3}\right)}^{-1}\right)}^{-1}}\\ & =256\times \frac{18.2k}{115k}\times \frac{1}{100k({\left(10.5k\right)}^{-1}+s\cdot 10n+{\left(18.2k+115k\right)}^{-1})+1}\\ & =3.594\times \frac{1}{\frac{s}{11274.56}+1} \end{array}$$

Substituting (53) and (54) into (55), the transfer function of the uncompensated loop gain equation can be obtained. The magnitude and phase at 1 kHz can be obtained by setting the crossover frequency to 1 kHz as follows:

(55) $$T_{u0}(s)=\frac{H(s)\cdot G_{vd}(s)}{V_{m}}=\frac{5.687\times 10^{4}}{\;6.925\times 10^{-9}s^{3}+3.689\times 10^{4}s^{2}+28.06s+2.794\times 10^{5}}$$

(56) $$|T_{u0}(j2\pi \cdot 1000)|=-14.9377\mathrm{dB},\;∠T_{u0}(j2\pi \cdot 1000)=-33.4439{}^{\circ}$$

According to the rule of thumb, the general condition for a stable system is that the phase PM is located between 45° and 75°. Therefore, the compensating phase is set to 60°. In (56), the phase that needs to be compensated by the PI controller at 1 kHz is shown below:

(57) $$60^{{}^{\circ}}=\theta_{PI}-33.4439{}^{\circ}-(-180^{{}^{\circ}})\Rightarrow \theta_{PI}=60^{{}^{\circ}}-180^{{}^{\circ}}+33.4439{}^{\circ}=-86.5561{}^{\circ}$$

From (56) and (57), the magnitude and phase of the transfer function of the PI controller at 1 kHz can be determined to calculate the proportional gain of the time-domain system, as shown in (58) and (59), respectively:

(58) $$G_{PI}(j2\pi \cdot 1000)=5.5832∠-86.5561{}^{\circ}=0.3353-j5.5731$$

(59) $$G_{PI}(j2\pi \cdot 1000)=K_{P}+\frac{K_{i}}{j2\pi \cdot 1000}\Rightarrow \left\{ \begin{array}{l} K_{p}=0.3353\\ K_{i}=35016 \end{array}\right.$$

The digital PI controller uses the discrete-time system to approximate the time-domain system by quickly sampling the values and summing up the errors. In the case of digital controllers, as mentioned in [29], the data delay affects the phase margin in the high-frequency region, and the sampling frequency determines the Nyquist Frequency. The Nyquist Frequency is the lowest frequency that suppresses aliasing. Accordingly, the sampling frequency *f_sample_* is set to be four times the switching frequency *f_s_*, i.e., the sampling frequency *f_sample_* is 234.4 kHz.

The proportional gain *K_pz_* and integral gain *K_iz_* of the digital PI controller use 8-bit precision, i.e., 1 over 256. By adjusting the gain *K_pz_* and *K_iz_* so that the loopback compensation capability of the digital PI controller is equivalent to that of the PI controller of the continuous time system, the relationships between the gains *K_p_* and *K_i_* of the continuous time system and the gains *K_pz_* and *K_iz_* of the discrete-time system are as follows:

(60) $$\left\{ \begin{array}{l} K_{p}=0.3353=3\times \frac{K_{pz}}{256}\\ K_{i}=35016=3\times \frac{K_{iz}}{256}\times f_{sample} \end{array}\right.$$

In practice, the gains are fine-tuned, and the resulting gains are set to $K_{pz}=50$ and $K_{iz}=12$.

## 6. Simulated and Experimental Results

After the design parameters of the proposed inverter are put into the circuit constructed by the simulation software PSIM 9.11 to simulate and verify the feasibility of the closed-loop control, the FPGA is used as the digital control kernel to measure some waveforms and data to demonstrate the effectiveness of the design.

**Figure 15:**
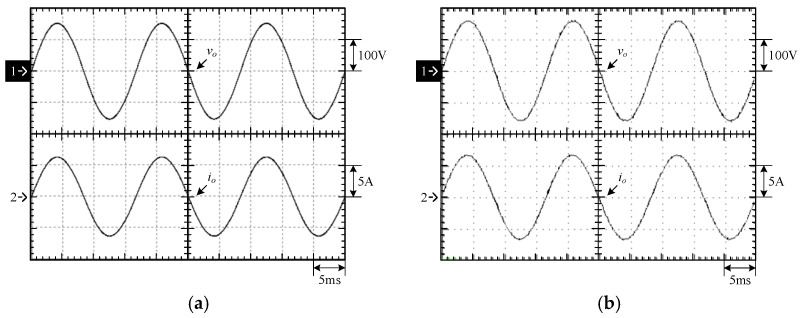
Output voltage and output current under 100% load: (1) *v_o_*; (2) *i_o_*. (**a**) Simulated waveforms, (**b**) Experimental waveforms.

**Figure 16:**
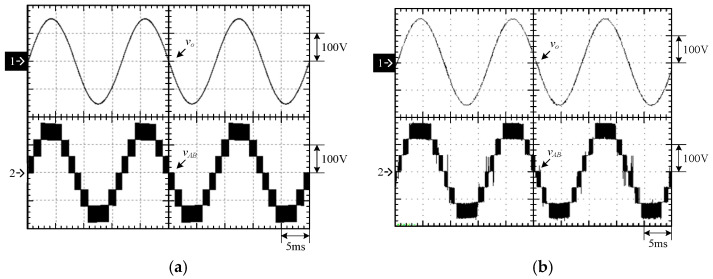
Output voltage and output current under 100% load: (1) *v_o_*; (2) *v_AB_*. (**a**) Simulated waveforms, (**b**) Experimental waveforms.

**Figure 17:**
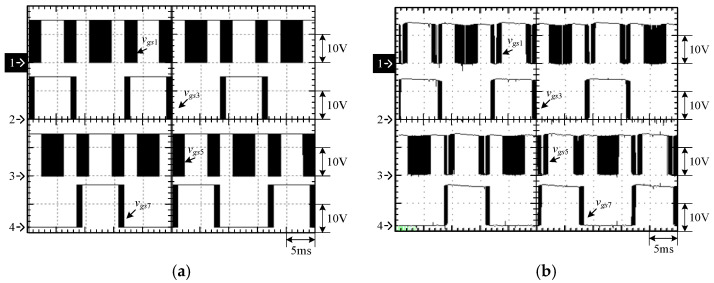
Gate driving signals for *S*_1_, *S*_3_, *S*_5_, and *S*_7_ under 100% load: (1) *v_gs_*_1_; (2) *v_gs_*_3_; (3) *v_gs_*_5_; and (4) *v_gs_*_7_. (**a**) Simulated waveforms, (**b**) Experimental waveforms.

**Figure 18:**
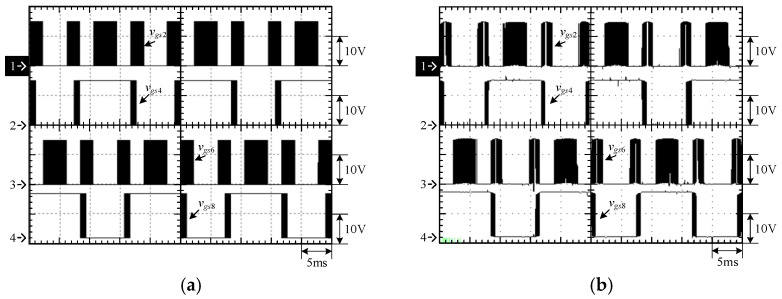
Gate driving signals for *S*_2_, *S*_4_, *S*_6_, and *S*_8_ under 100% load: (1) *v_gs_*_2_; (2) *v_gs_*_4_; (3) *v_gs_*_6_; and (4) *v_gs_*_8_. (**a**) Simulated waveforms, (**b**) Experimental waveforms.

**Figure 19:**
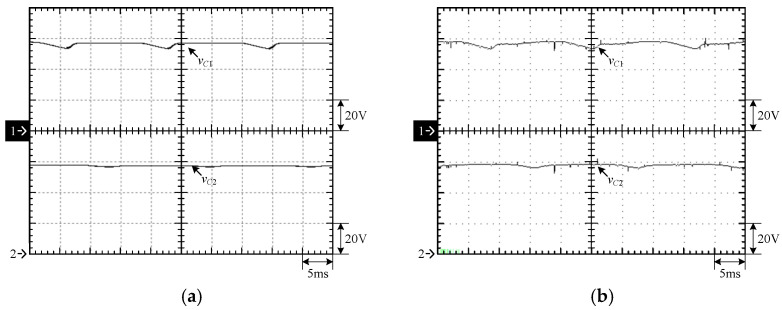
Voltages on *C*_1_ and *C*_2_ under 100% load: (1) *v_C_*_1_; (2) *v_C_*_2_. (**a**) Simulated waveforms, (**b**) Experimental waveforms.

**Figure 20:**
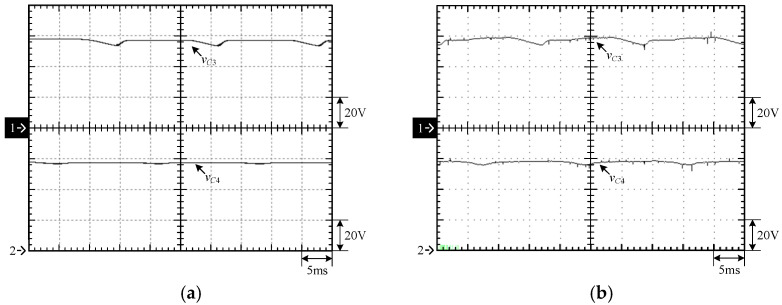
Voltages on *C*_3_ and *C*_4_ under 100% load: (1) *v_C_*_3_; (2) *v_C_*_4_. (**a**) Simulated waveforms, (**b**) Experimental waveforms.

**Figure 21:**
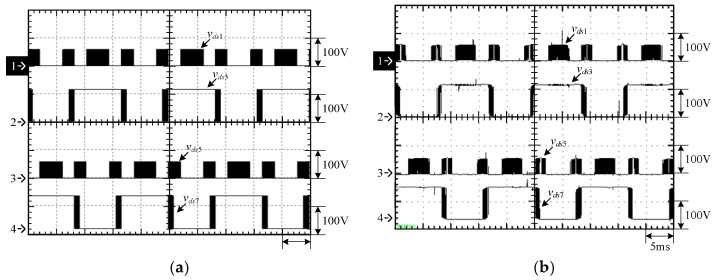
Voltages across *S*_1_, *S*_3_, *S*_5_, and *S*_7_: (1) *v_ds_*_1_; (2) *v_ds_*_3_; (3) *v_ds_*_5_; and (4) *v_ds_*_7_. (**a**) Simulated waveforms, (**b**) Experimental waveforms.

**Figure 22:**
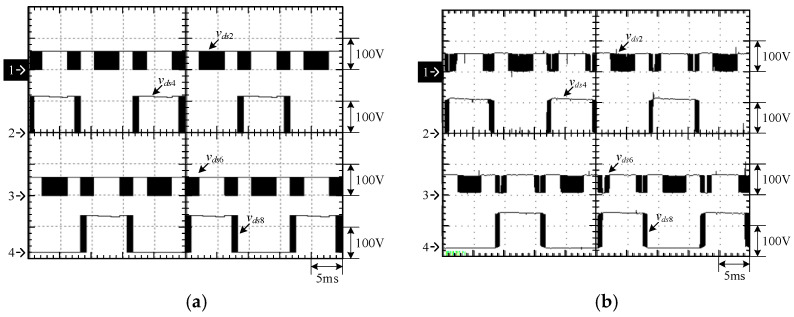
Voltages across *S*_2_, *S*_4_, *S*_6_, and *S*_8_: (1) *v_ds_*_2_; (2) *v_ds_*_4_; (3) *v_ds_*_6_; and (4) *v_ds_*_8_. (**a**) Simulated waveforms, (**b**) Experimental waveforms.

Figure 15 shows the output voltage *v_o_* and output current *i_o_* at 100% load. Figure 16 shows the output voltage *v_o_* and unfiltered output voltage *v_AB_* at 100% load. Figure 17 shows the gate driving signals *v_gs_*_1_, *v_gs_*_3_, *v_gs_*_5_, *v_gs_*_7_ for the switches *S*_1_, *S*_3_, *S*_5_, *S*_7_ under 100% load. Figure 18 shows the gate driving signals *v_gs_*_2_, *v_gs_*_4_, *v_gs_*_6_, *v_gs_*_8_ for the switches *S*_2_, *S*_4_, *S*_6_, *S*_8_ operating at 100% load. Figure 19 shows the clamping capacitors *C*_1_ and *C*_2_ operating at 100% load with cross-voltages *v_C_*_1_ and *v_C_*_2_. Figure 20 shows the clamping capacitance *C*_3_ and *C*_4_ at 100% load and the cross-voltages *v_C_*_3_ and *v_C_*_4_. Figure 21 shows the voltages *v_ds_*_1_, *v_ds_*_3_, *v_ds_*_5_, *v_ds_*_7_ on the switches *S*_1_, *S*_3_, *S*_5_, *S*_7_ under 100% load. Figure 22 shows the voltages *v_ds_*_2_, *v_ds_*_4_, *v_ds_*_6_, *v_ds_*_8_ on the switches *S*_2_, *S*_4_, *S*_6_, *S*_8_ operating at 100% load.

From the waveforms in Figure 15, Figure 16, Figure 17 and Figure 18, it can be known that the simulated results are quite consistent with the experimental results, and the converter generates the gate driving signal of the control switch through the positive and negative half-cycles symmetrically, making the switches operated at high frequency yields the unfiltered output voltage *v_AB_*, which is then passed through the second-order low-pass filter to obtain the low-harmonic output voltage *v_o_* and output current *i_o_*. In addition, by comparing the measured waveforms in Figure 17 and Figure 18 with the simulated waveforms, it can be shown that due to the consideration of the non-ideal characteristics of the power switch in practice, it is necessary to add the blanking time in the clamping half-bridge and output the half-bridge to avoid short-through. However, when switching from one stage to another, if the proportion of the switching period controlled in that stage is too small, the switching behavior of the power switch is ignored by the blanking time, and the waveforms show that the gate driving signal of the power switch is normally cutoff for part of the switching period. Figure 19 shows the clamping capacitors *C*_1_ and *C*_2_, which are switched by the diodes *D*_1_ and *D*_2_, and the switches *S*_1_ and *S*_2_ to clamp the voltages on *C*_1_ and *C*_2_ at the input voltage. Figure 20 shows the clamping capacitors *C*_3_ and *C*_4_, which are switched by the diodes *D*_3_ and *D*_4_, and the switches *S*_5_ and *S*_6_ to clamp the voltages on *C*_3_ and *C*_4_ at the input voltage. As shown in Figure 19 and Figure 20, the clamping capacitor cross-voltage of the double-voltage clamping circuit is the input voltage, and the simulated results are quite consistent with the experimental results. From Figure 21 and Figure 22, it can be shown that not only are the simulated results quite consistent with the experimental results but also the maximum clamping voltages of the switches *S*_3_, *S*_4_, *S*_7_, and *S*_8_ are the sum of the two clamping capacitor cross-voltages, i.e., twice the input voltage, whereas the switches *S*_1_, *S*_2_, *S*_5_, and *S*_6_ are all clamped at the input voltage, so they only need to withstand double the input voltage.

**Figure 23:**
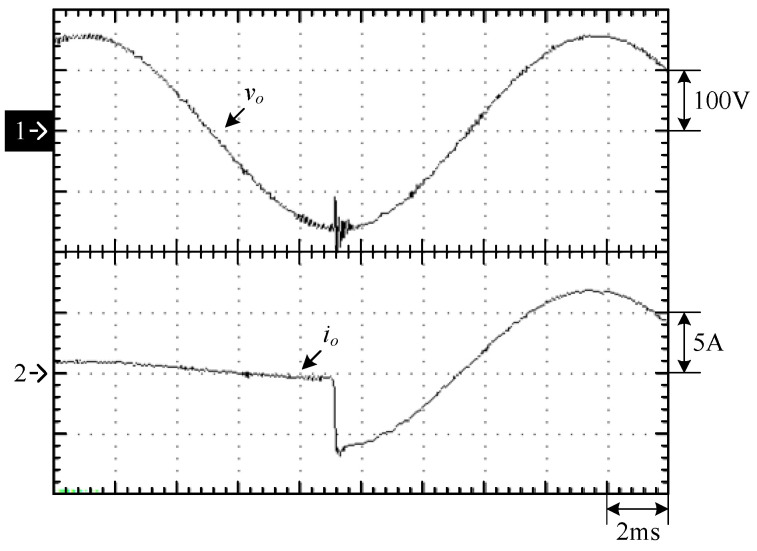
Experimental waveforms close to the peak of the negative half-cycle from the 10% load to 100% load: (1) *v_o_*; (2) *i_o_*.

**Figure 24:**
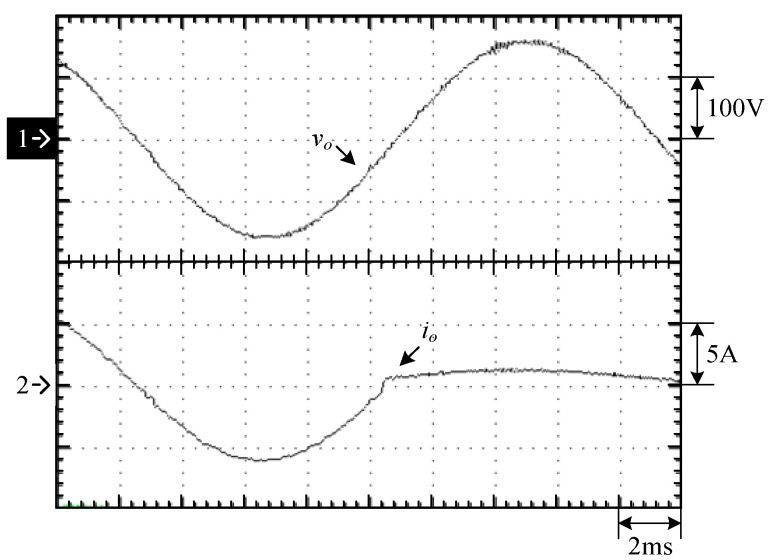
Experimental waveforms close to the zero-crossing point from 100% load to 10% load: (1) *v_o_*; (2) *i_o_*.

**Figure 25:**
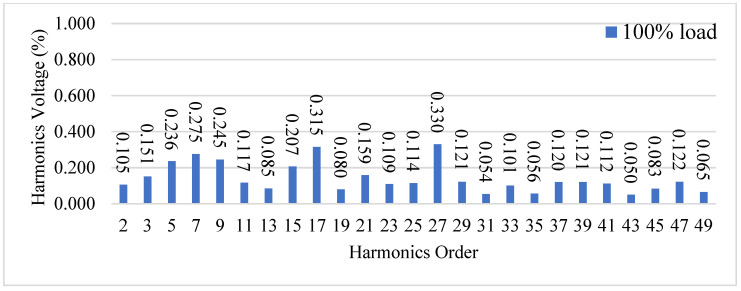
Output voltage harmonics under 100% load.

**Figure 26:**
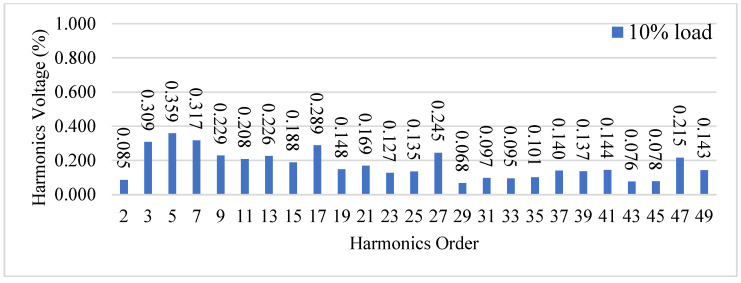
Output voltage harmonics under 10% load.

Figure 23 shows the waveforms of the output voltage *v_o_* and output current *i_o_* from the 10% load to 100% load. Figure 24 shows the waveforms of the output voltage vo and output current *i_o_* from 100% load to 10% load. Figure 23 shows that when the output voltage is close to the peak of the negative half-cycle, the output voltage and current can still be stabilized after uploading, and the corresponding recovery time is about 1 ms, and the corresponding output voltage drops by about 50 V due to a larger change in the output current. Figure 24 shows when the output voltage is close to the zero-crossing point, *v_o_* and *i_o_* can be stabilized from 100% load to 10% load. Since *i_o_* has a smaller variation, a change in *v_o_* is less clear. In addition, Figure 25 shows the output voltage harmonics under 100% load, whereas Figure 26 shows the output voltage harmonics under 10% load.

**Figure 27:**
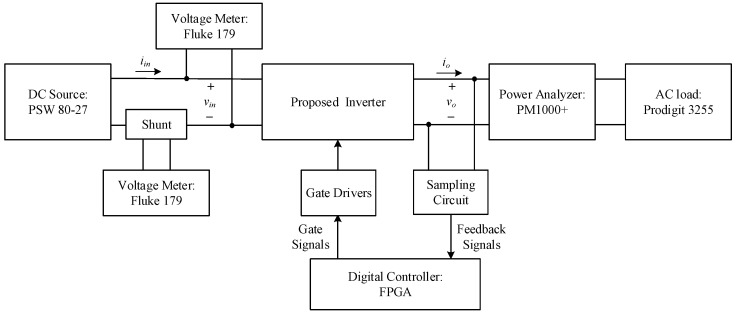
Block diagram for efficiency measurement.

Figure 27 shows the efficiency measurement method, where the current detection resistor (Shunt) is used to obtain the input current; two digital meters (Fluke179) are used to detect the voltage on the current detection resistor and to obtain the average value of the input current and the average value of the input voltage. Accordingly, the corresponding average input power can be calculated. In addition, an AC electronic load (Prodigit 3255) is utilized on the load side, and a power analyzer (PM1000+) is adopted to measure the output current, voltage, average output power, total harmonic distortion, and harmonic magnitude of each order. Finally, the resulting input and output powers are employed to calculate the efficiency of the actual circuit operation.

**Figure 28:**
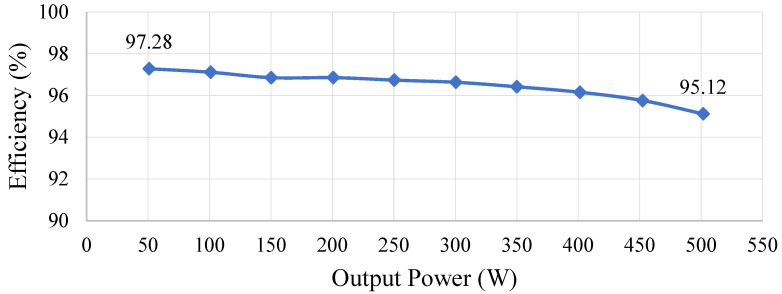
Curve of efficiency versus output power.

Figure 28 shows the efficiency curve of the proposed circuit. From this figure, the maximum efficiency of the proposed circuit is 97.28%, and the minimum efficiency is 95.11%. Since the converter is constructed using two double voltage clamping circuits connected symmetrically, the switches have low-voltage stress. As a result, the switches are characterized by low-voltage stress for better switching features. However, the circuit needs to store and release energy through the clamping capacitors to achieve a high-voltage gain. Consequently, the series of parasitic resistance (ESR) leads to additional energy loss during the operation period, resulting in a negative correlation between efficiency and output power, i.e., the higher the output power is, the lower the efficiency.

## 7. Conclusions

In this study, a single-phase, multilevel symmetric switched-capacitor inverter based on the concept of the double voltage clamping circuit connected to the half-bridge circuit was developed. Only one switch operated at any time, so not only was the corresponding control quite easy but also every switch had a very low switching loss and voltage stress. Furthermore, the proposed seven-level inverter was analyzed in detail by the operating principle as well as the mathematical model for the adopted level-shift sinusoidal pulse width modulation (LS-SPWM), which for this multilevel inverter was successfully developed using the well-known state averaging technique widely employed in the DC–DC converter. As a result, the required proportional-integral (PI) controller used in the closed loop can be designed systematically and easily. In addition, the overall efficiency is above 95.11% and up to 97.28%. At the same time, the total harmonic distortion (THD) under 100% load and 10% load are 0.46% and 0.48%, respectively. In addition, in order to meet a higher switching frequency, the switches were realized herein using MOSFETs. However, in motor drive applications, these MOSFETs can be substituted with IGBTs to obtain a lower switching frequency so as to reduce the switching loss as well as the electromagnetic interference (EMI).

## Figures and Tables

**Table 1:** Switching behavior and clamping capacitor charging and discharging behavior.

States	Switches								Capacitors				*v_AN_*	*v_BN_*	*v_AB_*
	*S*_1_	*S*_2_	*S*_3_	*S*_4_	*S*_5_	*S*_6_	*S*_7_	*S*_8_	*C*_1_	*C*_2_	*C*_3_	*C*_4_			
I	1	0	0	1	1	0	0	1	---	C	---	C	0 *V_in_*	0 *V_in_*	0 *V_in_*
II	0	1	1	0	1	0	0	1	C	---	---	C	1 *V_in_*	0	1 *V_in_*
III	1	0	1	0	1	0	0	1	D	---	---	C	2 *V_in_*	0	2 *V_in_*
IV	1	0	1	0	0	1	0	1	D	---	---	D	2 *V_in_*	−1 *V_in_*	3 *V_in_*
V	1	0	0	1	0	1	1	0	---	C	---	C	0 *V_in_*	1 *V_in_*	−1 *V_in_*
VI	1	0	0	1	1	0	1	0	---	C	---	C	0 *V_in_*	2 *V_in_*	−2 *V_in_*
VII	0	1	0	1	1	0	1	0	---	---	D	D	−1 *V_in_*	2 *V_in_*	−3 *V_in_*

**Table 2:** Specifications and components of the proposed switched-capacitor inverter.

Specification/Component	Model Name/Value
DC Input Voltage (*V_in_*)	58 V
AC Output Voltage (*v_o_*)	110 V_rms_
Output Frequency (*f_line_*)	60 Hz
Rated Power (*P_o,rated_*)	500 W
Clamped Capacitors (*C*_1_ to *C*_4_)	3.3 mF/100 V
Output Filter Capacitor (*C_o_*)	1 μF/275 V (MKP)
Output filter Inductors (*L_o_*_1_ and *L_o_*_2_)	142 μH (Ring Core)
Switching Frequency (*f_s_*)	58.6 kHz

**Table 3:** Components used in the voltage sampling circuit.

Geometric Type	Component Symbol	Model Name/Value
Four-Channel OPA	OP	TLV2374
Dip Resistors	*R*_1_ and *R*_5_	100 kΩ
SMD 0805 Resistors	*R*_2_ and *R*_6_	115 kΩ
SMD 0805 Resistors	*R*_3_ and *R*_8_	18.2 kΩ
SMD 0805 Resistors	*R*_4_ and *R*_7_	10.5 kΩ
SMD 0805 Resistors	*R*_9_ and *R*_10_	2 kΩ
0805 MLCC	*C*_5_ and *C*_6_	10 nF

## Data Availability

Data are contained within the article.
